# A genome-wide association study of reproduction traits in four pig populations with different genetic backgrounds

**DOI:** 10.5713/ajas.19.0411

**Published:** 2019-10-21

**Authors:** Yao Jiang, Shaoqing Tang, Wei Xiao, Peng Yun, Xiangdong Ding

**Affiliations:** 1 National Engineering Laboratory for Animal Breeding, Laboratory of Animal Genetics, Breeding and Reproduction, Ministry of Agriculture, College of Animal Science and Technology, China Agricultural University, Beijing 100193, China; 2 Beijing Station of Animal Husbandry, Beijing 100107, China

**Keywords:** Genome-wide Association Study, Total Number Born, Number Born Alive, Meta-analysis

## Abstract

**Objective:**

Genome-wide association study and two meta-analysis based on GWAS performed to explore the genetic mechanism underlying variation in pig number born alive (NBA) and total number born (TNB).

**Methods:**

Single trait GWAS and two meta-analysis (single-trait meta analysis and multi-trait meta analysis) were used in our study for NBA and TNB on 3,121 Yorkshires from 4 populations, including three different American Yorkshire populations (n = 2,247) and one British Yorkshire populations (n = 874).

**Results:**

The result of single trait GWAS showed that no significant associated single nucleotide polymorphisms (SNPs) were identified. Using single-trait meta analysis and multi-trait meta analysis within populations, 11 significant loci were identified associated with target traits. Spindlin 1, vascular endothelial growth factor A, forkhead box Q1, msh homeobox 1, and LHFPL tetraspan submily member 3 are five functionally plausible candidate genes for NBA and TNB. Compared to the single population GWAS, single-trait Meta analysis can improve the detection power to identify SNPs by integrating information of multiple populations. The multiple-trait analysis reduced the power to detect trait-specific loci but enhanced the power to identify the common loci across traits.

**Conclusion:**

In total, our findings identified novel genes to be validated as candidates for NBA and TNB in pigs. Also, it enabled us to enlarge population size by including multiple populations with different genetic backgrounds and increase the power of GWAS by using meta analysis.

## INTRODUCTION

China produces more than 50 million tons of pork each year, accounting for over 50% of the total global production [1]. Even a slight increase in Chinese pork production will have a significant impact on the global pork market. Reproductive traits, such as total number born (TNB) and number born alive (NBA), have been considered as the most important index included in the selection indices of pig breeding programs for evaluating sow productivity [2]. Up to the present, selection based on traditional breeding methods using best linear unbiased prediction has been successful in improving maternal reproductive traits [3]. However, the genetic architecture of reproductive traits is very complicated due to low heritability, minor genes, maternal effects and environmental factors [4], resulting in the difficulty deciphering the genetic architecture of reproduction traits. Over the past 20 years, the dense genome coverage provided by high-throughput chip genotyping makes it possible to exploit the linkage disequilibrium (LD) between single nucleotide polymorphisms (SNPs) and quantitative trait locus (QTL) through genome-wide association study (GWAS) to identify genes related to traits of interest. Several GWAS have been revealed significant associations for economically important traits such as reproduction traits [5], growth traits [6], meat traits [7], and feed conversion [8].

Exploring the loci and genes affecting sow reproduction performance is necessary for understanding the genetic characteristics of these traits and increasing the speed of genetic improvement [9]. On one hand, genes such as estrogen receptor 1 [10], insulin-like growth factor 2 [11], and aryl hydrocarbon receptor [12] had been identified as important candidate genes positively associated with reproduction. But these genes explain only a relatively small proportion of the genetic variance. On the other hand, limited by the size of the population and other factors, only small number of genes were detected in association analysis. According to the pig QTLdb (https://www.animalgenome.org/cgi-bin/QTLdb/SS/index), until now, there are 2,135 QTLs reported for overall traits linked to reproduction including 6 QTLs for endocrine, 1,013 for litter traits, 668 for reproductive organs, and 448 QTLs for reproductive traits. Among them, 522 QTLs identified for TNB (350 QTLs) and NBA (172 QTLs) compared with 8,962 QTLs for fatness or 1,745 for growth traits.

Most of those studies mainly utilize one pure breed or an intercross population, with the result only reflecting one specific breed’s linkage disequilibrium character. As the most popular commercial pig breed, Yorkshire is usually used as terminal dam line in pig hybrid production, and the improvement on their reproduction traits is therefore very important. There were two main objectives in our study. The first one was to detect significant SNPs and candidate genes in four populations from different genetic backgrounds using conventional single-trait GWAS respectively. The second one is to improve the power of GWAS by enlarging population size through implementing a meta-analysis for multiple traits within a population or for same trait across populations.

## MATERIALS AND METHODS

### Ethics statement

The whole procedure for collecting ear tissue samples was carried out in strict accordance with the protocol approved by the Institutional Animal Care and Use Committee (IACUC) at the China Agricultural University. The IACUC of the China Agricultural University specifically approved this study (permit number DK996).

### Animals and phenotype

A total of 3,121 Yorkshire pigs used in this study were sampled from four pig breeding farms (abbreviated as LM, FJ, XD, ZX for convenience), including 2,247 progeny of three different American Yorkshire populations and 874 progeny of one British Yorkshire population (XD). Animals from LM and ZX are descendants of American Yorkshires but from different breeding companies, while LM and FJ came from the same breeding companies. The progeny of American Yorkshires were born in 2013 through 2018 and came from 222 sire families (8 to 76 offspring in each family with an average of 15), and the progeny of British Yorkshires were born in 2007 through 2013 and came from 129 sire families (10 to 71 offspring in each family with an average of 7). There was no genetic connectedness between LM, XD, and ZX according to the pedigree information. Phenotypic records included two reproductive traits, TNB and NBA. The populations and phenotypes information are presented in Table 1.

Breeding values for NBA and TNB were routinely estimated by the breeding companies using a standard animal repeatability model which was separately implemented in each population, and were obtained from the National Swine Genetic Improvement Center of China (http://cnsge.nahs.org.cn/); afterwards, corrected phenotypic values were calculated as EBV plus the estimated residual for each individual in each population.

### Genotyping and quality control

Genomic DNA was extracted from blood samples using a TIANamp Blood DNA Kit (catalog number DP348; Tiangen, Beijing, China). Genotyping was performed using a PorcineSNP80 BeadChip (Illumina, San Diego, CA, USA), which includes 68,528 SNP across the entire pig genome. Genotype quality control was carried out using PLINK 1.9 software [13] separately for each population. First, individuals with call rates (CR) less than 90% were removed and then SNP with CR less than 90%, minor allele frequencies <3%, or significant deviation from the Hardy–Weinberg equilibrium (p<10× 10^−6^) were removed. After genotype quality control, 3,121 individuals and 49,839 SNP remained for further analysis.

#### Population structure

Because the genetic backgrounds of four Yorkshire populations in this study are different, a principal component analysis (PCA) was carried out to detect the population stratification using GCTA software [14]. In order to keep the independence of SNPs, the adjacent SNPs with r^2^ greater than 0.2 were further pruned after genotype quality control, and in total 29,229 SNPs were used in PCA. The linkage disequilibrium within each population was calculated using PLINK software [13] as well. Meanwhile, a quantile-quantile (Q-Q) plot was generated to assess the influence of population stratification on the GWAS.

### Statistical analysis

Single-population GWAS through linear mixed model was carried out in each pig population separately. Based on single-population analysis, the meta-analysis within population and cross populations were conducted, respectively.

### Genome-wide association study for a single trait in a single-population

#### Linear mixed model

A linear mixed model was implemented to detect the association of SNP with growth and fatness traits. The model in this study is a single SNP regression model. The model includes a random polygenic effect to account for shared genetic effects of related individuals and to control population stratification. The statistical model is described below:

$$y_{c}=1\mu +bx+\mathrm{Zg}+e,$$

in which **y_c_** is the vector of phenotypes (corrected phenotypic values); **1** is a vector of ones; μ is the overall mean; *b* is the average effect of the gene substitution of a particular SNP; **x** is a vector of the SNP genotype (coded as 0, 1, or 2); **g** is a vector of random polygenic effects with a normal distribution **g** ~ *N*(0, **G**σ_a_^2^), in which σ_a_^2^ is the polygenic variance and **G** is the genomic additive relationship matrix and was constructed using all markers following VanRaden [15]; **Z** is an incidence matrix relating phenotypes to the corresponding random polygenic effects; and **e** is a vector of residual effects with a normal distribution *N*(0, **I**σ_e_^2^), in which σ_e_^2^ is the residual variance. The software GCTA [14] was used to fit the model.

Afterwards, Bonferroni correction at a significance level of 0.05 was used to identify significant SNP. There were 52,173, 52,804, 52,526, and 52,267 qualified SNPs in the four populations (LM, FJ, XD, and ZX), respectively. The p values of the 5% genome-wide and suggestive significant thresholds were equal to 0.05/SNPs number and 1/SNPs number, respectively, in four populations.

### Meta-analysis of GWAS for a single trait across populations (MS-GWAS)

Based on the results of GWAS separately in four populations through single-population analysis, and the meta-analysis based on Fisher’s method was carried out to combine P-value probabilities from each test into one test statistic (*X*^2^) using the formula:

$$X^{2}=-2\sum_{t=1}^{T}\text{ln}(P_{t}),$$

in which *p_i_* is the raw p-value of *t*th study for *t* = 1, …, *T*, in which *T* is the number of independent studies. When all the null hypotheses are true, this combined test statistic follows a χ^2^ distribution with 2*T* of degree of freedom. Therefore, the new p-value from the meta-analysis was calculated using:

$$P_{Fisher}=1-\text{Pr}(\chi_{2T}^{2}\leq X^{2}),$$

in which χ^2^_2T_ is a χ^2^ variable with 2*T* of degree of freedom. In our study, we used the common SNP in four population by Fisher’s method to calculate a meta-analysis p-value. Afterwards, Bonferroni correction at a significance level of 0.05 was used to identify significant SNP. There were 48,966 common SNPs in the four populations, the threshold p-value for each SNP at significance level of 0.05 was 1.02×10^−6^ (0.05/48,966).

### Meta-analysis of GWAS for multiple traits within population

In the present study, the traits NBA and TNB reflecting similar fertility function can be considered as different traits with some common genetic components. Therefore, the meta-analysis was performed within reproduction traits, TNB and NBA.

An approximated chi-square statistic [16] was applied to test whether there is at least one of the SNP effect of studied traits was not equal to zero. For each SNP, chi-square statistic of a multi-trait meta-analysis was calculated using the following formula:

$$\chi^{2}=t_{i}^{\prime }V^{-1}t_{i}$$

where *t_i_* was a vector of signed t-values of the *i*th SNP for all studied traits, $t_{i}^{\prime }$ was a transpose of the vector *t_i_*, V^−1^ was an inverse of the correlation matrix where the correlation between a pair of traits was estimated from the correlation of summary statistics over the SNPs in the analysis. Afterwards, Bonferroni correction at a significance level of 0.05 was used to identify significant SNP as same as genome-wide association study for a single trait in a single-population (SS-GWAS).

### Identification of candidate genes

To identify functionally plausible candidate genes near the significant SNP, the genes located in or overlapping the region between the 0.5 Mb upstream and 0.5 Mb downstream of the significant SNP were obtained using Ensemble (http://www.ensembl.org/Sus_scrofa/Info/Index; Sscrofa 11.1 genome version). Gene ontology analysis was carried out using the DAVID bioinformatics resource (https://david.ncifcrf.gov/). Pathway analysis was conducted using the online KEGG (http://www.kegg.jp/kegg/pathway.html) and GeneCards (http://www.genecards.org/) tools.

## RESULTS

### Population structure

**Figure 1:**
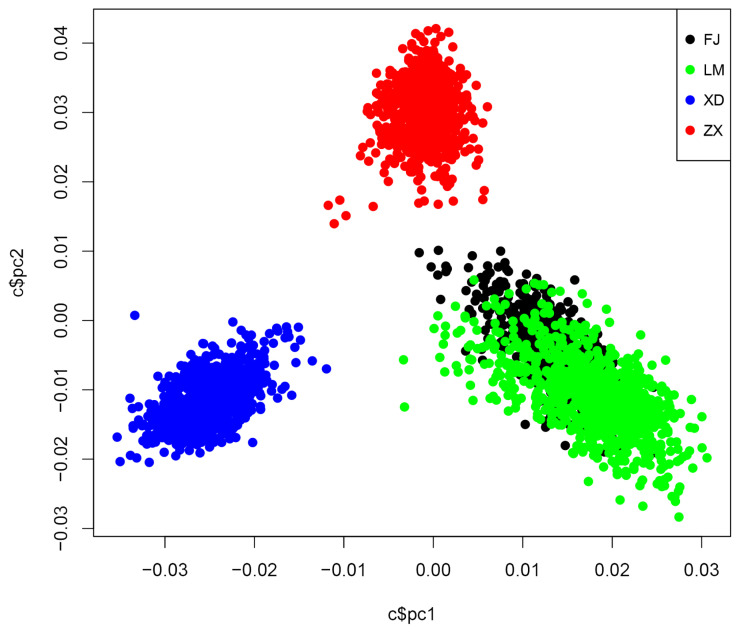
The statistics of population structure in 4 Yorkshire populations. PCA, principal component analysis for 4 Yorkshire populations. XD, FJ, LM, and ZX represent 4 Yorkshire populations from 4 elite Chinese pig breeding farms. pc1 = first principal component; pc2 = second principal component.

To identify the population structure of the four Yorkshire populations involved in this study, a PCA was performed using the chip data. As shown in Figure 1, the four Yorkshire populations from four farms can be clearly identified through PCA. The genetic backgrounds of the LM and FJ populations were classified nearly into one cluster implying no significant genetic differentiation among them. Meanwhile, both LM and FJ populations were divergent from ZX population, as they came from different American Yorkshires breeding companies. Likewise, XD was distantly related to FJ, LM, and ZX due to its British origins.

### SNPs identified by SS-GWAS for TNB and NBA in four populations

All significant SNPs associated with TNB and NBA traits in single population analysis are illustrated in Table 1. For SS-GWAS, the p values of the 5% (suggestive) genome-wide significant threshold were equal to 9.58×10^−7^ (1.92×10^−5^), 9.47×10^−7^ (1.89×10^−5^), 9.52×10^−7^ (1.90×10^−5^), 9.57×10^−7^ (1.91 ×10^−5^) in these four populations (LM, FJ, XD, and ZX), respectively. A total of 13 SNPs, of which six SNPs reached the genome-wide suggestive level for NBA and seven SNPs reached the genome-wide suggestive level for TNB, as shown in Table 2. Among that, six suggestive SNPs for XD, one for FJ, six for ZX and no SNPs for LM. The results of single population GWAS are in Supplementary Figure S1. No significant SNP was found in the single population GWAS results.

### SNPs identified by MS-GWAS for NBA and TNB across populations

**Figure 2:**
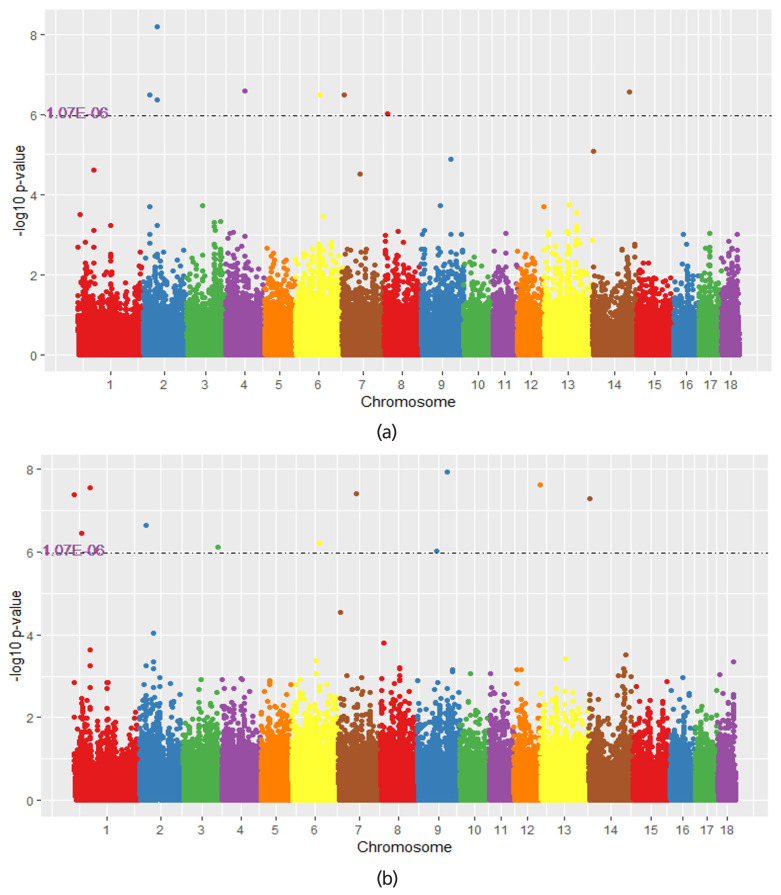
Manhattan plot of different analyses methods for total number born (TNB) and number born alive (NBA). The x-axis represents the chromosomes and the y-axis represents the −log_10_(p-value). The dotted line indicates the significance threshold for the (a) the result of MS-GWAS in the 4 populations for NBA, (b) the result of MS-GWAS meta-analysis in the 4 populations for TNB.

Manhattan plots for meta-analysis across populations are presented in Figure 2, while the summary of significant SNPs for TNB and NBA in the meta-analysis across populations is listed in Table 3. In total, 19 significant SNPs were detected for the traits analyzed in the meta-analysis across populations: 11 for TNB and 8 for NBA. Among them, 5 significant SNPs which were detected in TNA or NBA were also reached a suggestive level in another trait. In addition, the number of significant SNPs identified in meta analysis across populations were larger and more significant than in single-trait analysis. Ten suggestive SNPs which detected in SS-GWAS reached the higher level of significance in meta analysis across populations. Besides that, 11 SNPs that were significant in meta analysis across populations became more significant in multi-traits meta in the next analysis.

### SNPs identified by MM-GWAS within population

**Figure 3:**
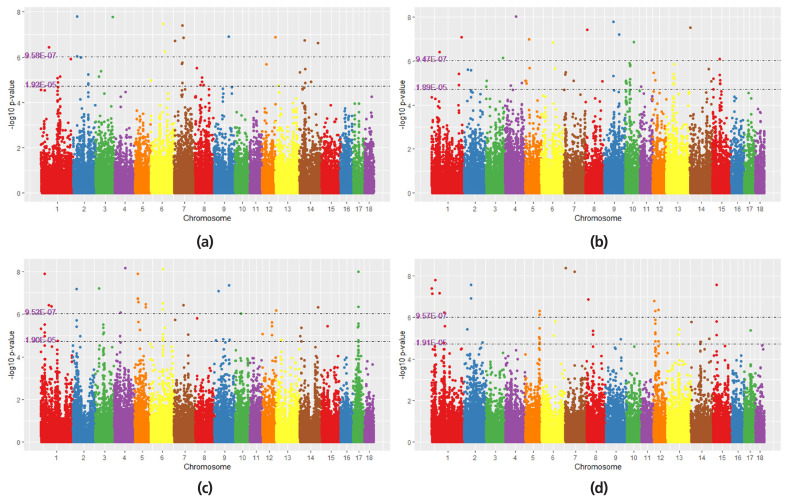
Manhattan plot of GWAS for multi-traits meta analysis within four populations. (a) the result of Meta-analysis of multiple traits within LM population; (b) the result of Meta-analysis of multiple traits within FJ population; (c) the result of Meta-analysis of multiple traits within XD population; (d) the result of Meta-analysis of multiple traits within ZX population. The x-axis represents the chromosomes and the y-axis represents the −log_10_(p-value). The first dotted line indicates the significance threshold of genome-wide level for multi-traits meta analysis. The second dotted line indicates the significance threshold of chromosome-wide level for multi-traits meta analysis.

Genomic correlation matrices between TNB and NBA were constructed in four populations, respectively. The absolute of genomic correlation was 0.89, 0.85, 0.87, and 0.89 for each population of LM, FJ, XD, and ZX, respectively. Manhattan plot for MM-GWAS within four populations are presented in Figure 3. In LM population (Figure 3a), the MM-GWAS revealed 42 SNPs associated with both TNB and NBA, including 13 with genome-wide significance and 29 with suggestive significant level. There were 60 SNPs identified for FJ population (Figure 3b), including 13 genome-wide significant loci and 47 genome-wide suggestive loci. For XD population (Figure 3c), 24 reached the significant level and 42 reached suggestive significant level. For ZX population (Figure 3d), 16 significant and 35 suggestive loci were identified. Compared the results of four populations, only 11 SNPs reached the significant or suggestive significant level in each population (Table 4). Besides that, these 11 significant/suggestive SNPs were all overlapped in the result of meta-analysis of GWAS (MS-GWAS). The significant/suggestive SNPs detected using the multi-trait meta-analysis within population are listed in Table 4.

### Identification of candidate genes

Based on 11 common significant SNPs associated with TNB and NBA identified by two meta-analysis methods, 11 genes which located within the region between the 0.5 Mb upstream and 0.5 Mb downstream of the significant/suggestive SNP were found and annotated (Tables 2 to 4). While Go ontology analysis revealed that, there were five annotated genes had a highlight biology function with TNB and NBA. All these annotated genes were selected based on the Sus scrofa 11.1 genome assembly. Further function annotation was carried on based on the NCBI database (https://www.ncbi.nlm.nih.gov/).

### Quantitative traits locus overlapped with SNPs

Until now, there were 522 pig QTLs identified for reproduction traits TNB (350 QTLs) and NBA (172 QTLs) in the pig QTL database (https://www.animalgenome.org/cgi-bin/QTLdb/SS/index). After comparing these QTL with the regions of 11 common significant SNPs, 6 SNPs were identified located in 5 QTLs which were identified before in Sus scrofa chromosome 6 (SSC6), SSC7, and SSC9 for TNB and NBA traits (https://www.animalgenome.org/cgi-bin/QTLdb/SS/traitmap?trait_ID=157 or trait_ID=156). This implies the functional genes such as msh homeobox 1 (*MSX1*), vascular endothelial growth factor A (*VEGFA*), forkhead box Q1 (*FOXQ1*), and LHFPL tetraspan submily member 3 (*LHFPL3*) around these SNPs are likely candidates for TNB and NBA traits.

## DISCUSSION

According to the results of genes annotated, a total of 11 genes of which five are relevant with two traits of reproduction, these candidate genes could regulate or influence TNB and NBA through different kinds of biological processes and pathways. *MSX1*, spindlin 1 (*SPIN1*), *VEGFA*, *FOXQ1*, and *LHFPL3* are highlighted as promising biological candidate genes for reproduction traits. Except *MSX1* and *VEGFA*, *SPIN1*, *FOXQ1*, and *LHFPL3* are reported as related to reproduction in pigs for the first time. MSX1 is associated with the utero embryonic development and some classical pathways in embryo development such as Wnt/Hedgehog/Notch pathway. Daikoku et al [17] and Nallasamy et al [18] found that *Msx1* was expressed in the preimplantation mouse uterus, and are critical for fertility in mice. Cha et al’s research [19] suggested that these transcription factors have cell-specific functions in the pregnant uterus, and its subsequent morphological and functional changes. *MSX1* had been already reported relevant with NBA and TNB in pigs [20]. *SPIN1* is necessary for normal meiotic progression in mammals [21]. In a previous study, Choi et al [22] revealed that *SPIN1* may play an important role in meiosis II (MII) arrest as well as in the regulation of early embryonic development. As to *VEGFA*, has key function in uteroplacental vasculogenesis during embryonic implantation and provides the vascular network to the placenta [23]. In previous pig GWAS studies, the *VEGFA* gene has been reported to be associated with TNB and NBA [20,24], and our study confirmed the previous investigations. Also, *LHFPL3*, a member of the family of LHFP-like genes. A direct link between *LHFPL3* and reproductive traits has not been reported before, but Ptacek et al [25] reported that it was closely related to uterine leiomyoma and highly expressed in the uterus. As we all known, uterus is important to embryonic development. So, *LHFPL3* might have a key function in conceiving and maintaining pregnancy. *FOXQ1* has not been defined in pigs, whereas in mice, it is involved in patterning the early embryonic mesoderm [26] and expressed at embryo day 8.5 [27]. Our findings will be helpful for a better understanding of the role of *FOXQ1* for embryo development in pig reproduction.

Population stratification is a major factor in false positives in GWAS for significant SNPs [28]. At present, there are 4 popular methods to resolve the problem of population stratification, genomic control, structured association, PCA, and linear mixed model which can deal with population stratification by taking polygenic effects into account. In general, a genomic inflation factor λ of <1.05 indicates no population stratification [29]. Our values were 1.788 to 3.24 for TNB and NBA in four populations before using genomic relationship matrix G. According to Janss et al [30], inclusion of principle components in model to account for population structure is not required, mainly because of the genetic variation accounted for after the genomic relationship matrix G has been considered in the model. Thus, PCA is incorporated in individual population analyses. Here, population stratification was adjusted through a constructed relationship matrix using genotype data in the linear mixed model. Genomic relationship can more accurately reflect the actual existing relationship between animals compared to pedigree-based relationships. By using G matrix (genomic additive relationship matrix) instead of A matrix (additive genetic relationship matrix), genomic inflation factor λ reduced to 1.02 to 1.04 for TNB and NBA which implied the genomic relationship matrix adequately controlled population stratification, as the Q-Q plot indicated (Supplementary Figure S2). This made the GWAS results more reliable.

In our study, two meta-analysis were conducted to explore the genomic loci for TNB and NBA based on the results of single trait GWAS in four pig populations. The results of GWAS for a single trait in a single population showed that no common significant SNPs were detected in the four populations (Table 1, Supplementary Figure S1) for TNB and NBA. Most of suggestive SNPs detected in XD did not appear in the ZX population. Only two SNPs (WU_10.2_4_80076056 and WU_10.2_1_11153176) were repeated in FJ and XD. These observations further reflected the complex genetic architecture of pig reproduction traits. The low consistency of findings from single population is similar with other investigations in GWAS. Many researches [31] performed a GWAS on different pig populations and revealed SNPs and candidate genes related target traits. But no overlapped significant results were identified in multiple populations. A possible reason for lack of genome-wide significant SNP is that small sample size, different population structures or the complexity of traits [32]. Liu’s et al research [33] confirmed that a large population size is important for GWAS in traits with low heritability. Combining different populations could reveal hidden or unclear associations that may not be detected by an independent study [32]. Novel significant SNPs could be detected in GWAS due to the greater power with increased sample size [34]. Our results indicated that meta-analysis could be efficient tool to improve the power of GWAS by combining different populations. Moreover, meta-analysis can increase statistical power especially for a locus with small effect by collectively using information from multiple independent studies [34].

No locus was identified significant by single population GWAS for TNB and NBA, with most significant SNPs detected across the two meta analysis approaches. In addition, compared to GWAS, 11 novel significant SNPs were detected using meta-analysis. Besides that, meta-analysis made the p-value smaller and more significant which were also consistent with Guo et al [35] and Le et al [36].

Significant SNPs were not found in single population GWAS, which showed the enhanced capacity of multi-trait approaches for detecting SNPs, particularly when the phenotypes are correlated [37]. Our finding that SNPs, which cannot be detected at a genome-wide significance level in GWAS, can be uncovered in a multi-trait approach corroborates the findings of Willer et al [32]. The joint association analysis of multiple phenotypes might be a more powerful approach to detect SNPs that underlie correlated traits than the multi-trait test statistic applied in our study [38].

In summary, we conducted GWAS separately in four Yorkshire populations, which have distinct genetic backgrounds, to identify genomic loci for reproduction traits of TNB and NBA. Based on results of GWAS, two kinds of Meta-analysis were implemented to improve the power of gene identification. Compared to the single population GWAS, single-trait meta analysis can improve the detection power to identify SNPs in a larger multiple population. The multiple-trait analysis reduced the power to detect trait-specific loci but enhanced the power to identify the common loci across traits. In total, 11 significant loci were identified associated with target traits. *SPIN1*, *VEGFA*, *FOXQ1*, *MSX1*, and *LHFPL3* are five functionally plausible candidate genes for TNB and NBA. Our findings further revealed that the meta-analysis and the multi-trait method can be used to increase the power of GWAS to identify genes relevant with important traits of interest.

## Figures and Tables

**Table 1:** Descriptive statistics of number of piglets born alive and total number of piglets born in 4 Yorkshire populations

Trait	Population1)	Source	N	Min	Max	Mean	SD
NBA	LM	American line2)	931	5.46	12.78	9.30	2.112
	FJ		545	6.60	14.38	10.70	2.295
	XD	British line	874	7.54	12.28	10.38	1.733
	ZX	American line3)	771	7.18	13.19	9.74	1.914
TNB	LM	American line2)	931	6.15	14.38	10.03	2.226
	FJ		545	6.89	16.75	11.73	2.489
	XD	British line	874	8.06	13.59	10.88	1.810
	ZX	American line3)	771	7.62	15.00	11.04	2.155

NBA, number of piglets born alive; TNB, total number of piglets born; SD, standard deviation.

1) LM, FJ, XD, and ZX are Yorkshire populations from 4 elite Chinese pig breeding farms.

2),3) Animals from LM, FJ, and ZX are descendants of American Yorkshires but ZX was came from another breeding company.

**Table 2:** The result of single population analysis for total number of piglets born and number of piglets born alive traits across populations

SSC	SNP name	Location (bp)	Populations1)	p_(NBA)_-value2)	p_(TNB)_-value3)	Associated gene4)	Distance5) (bp)	Gene function
17	ALGA0094112	27,884,015	XD	**1.160E-05**	3.770E-05	*RIN2*	In	Signal transduction
1	WU_10.2_1_11153176	9,032,844	XD	**1.686E-05**	3.314E-05	*SYNJ2*	In	Inositol phosphate dephosphorylation
6	MARC0081527	80,617,330	XD	**1.859E-05**	1.780E-05	*C1QB*	Down	Complement activation, classical pathway
4	WU_10.2_4_80076056	73,428,123	XD	2.188E-05	**1.336E-05**	*CA8*	Down	Phosphatidylinositol-mediated signaling
2	ALGA0113046	12,842,137	XD	2.132E-05	**1.346E-05**	***OR10Q1***	Up	G protein-coupled receptor signaling pathway
5	H3GA0015463	9,863,427	XD	3.220E-05	**1.760E-05**	*PICK1*	In	Intracellular protein transport
4	WU_10.2_4_80076056	73,428,123	FJ	**1.807E-05**	2.313E-05	*CA8*	Down	Phosphatidylinositol-mediated signaling
12	WU_10.2_12_17971455	41,032	ZX	**1.080E-05**	7.546E-04	*ZNF750*	Down	
7	WU_10.2_7_130172562	537,474	ZX	**1.675E-05**	1.040E-05	***FOXQ1***	Up	Cell differentiation
1	WU_10.2_1_11153176	9,032,844	ZX	6.517E-05	**1.110E-05**	*SYNJ2*	In	Inositol phosphate dephosphorylation
7	H3GA0021245	38,823,945	ZX	2.478E-05	**1.125E-05**	***VEGFA***	Down	Regulation of signaling receptor activity
1	MARC0022141	0	ZX	1.172E-04	**1.644E-05**	NA	NA	NA
1	DRGA0000439	30,456,989	ZX	1.918E-04	**1.674E-05**	*EYA4*	In	Protein dephosphorylation

SSC, Sus scrofa chromosome; SNP, single nucleotide polymorphism; NBA, number of piglets born alive; TNB, total number of piglets born; *RIN2*, Ras and Rab interactor 2; *SYNJ2*, synaptojanin 2; *C1QB*, complement C1q B chain; *CA*, carbonic anhydrase 8; *OR10Q1*, olfactory receptor family 10 subfamily Q member 1; *PICK1*, protein interacting with PRKCA 1; *ZNF750*, zinc finger protein 750; *FOXQ1*, forkhead box Q1; *VEGFA*, vascular endothelial growth factor A; *EYA4*, EYA transcriptional coactivator and phosphatase 4; NA, not available; GWAS, genome-wide association study.

1) The results of significant SNPs from different populations.

2) p_(NBA)_–value = p-value from sing population GWAS for NBA.

3) p_(TNB)_–value = p-value from sing population GWAS for TNB. The bold data in this column represent the SNP at suggestive genome-wide significant level.

4) The associated gene in bold in this column represent these genes were associated with traits based on annotation.

5) down/up = the location of SNP in downstream/upstream of the nearest gene.

**Table 3:** The results of meta-analysis for total number of piglets born and number of piglets born alive traits across populations

SNP name	SSC	Location (bp)	p_(meta-TNB)_–value1)	p_(meta-NBA)_–value2)	Associated gene3)	Distance4) (bp)	Gene function
ALGA0012964	2	32,799,355	9.23E-05	**6.41E-09**	*LIN7C*	In	Morphogenesis of an epithelial sheet
ALGA0054421	9	104,625,702	**1.17E-08**	1.29E-05	***LHFPL3***	In	Self reported educational attainment
ASGA0037579	8	5,703,865	0.000156302	**9.82E-07**	***MSX1***	Down	In utero embryonic development
ASGA0082366	9	47,037,744	**9.84E-07**	0.000189917	*NECTIN1*	Down	Lens morphogenesis in camera-type eye
ALGA0113046	2	12,842,137	**2.29E-07**	0.000193931	*OR10Q1*	Up	G protein-coupled receptor signaling pathway
ASGA0104976	12	116,492,539	**2.44E-08**	0.000203328	NA		
DRGA0000439	1	30,456,989	**2.86E-08**	2.37E-05	*EYA4*	In	Protein dephosphorylation
H3GA0042513	14	126,349,106	0.000299386	**2.80E-07**	*GFRA1*	In	Nervous system development
WU_10.2_7_130172562	7	537,474	2.88E-05	**3.27E-07**	***FOXQ1***	Up	Cell differentiation
H3GA0021245	7	38,823,945	**3.95E-08**	3.11E-05	***VEGFA***	Down	Regulation of signaling receptor activity
MARC0022141	1	0	**4.11E-08**	0.000318796	NA	NA	NA
WU_10.2_4_80076056	4	73,428,123	0.00116	**2.62E-07**	*CA8*	Down	Phosphatidylinositol-mediated signaling
WU_10.2_1_11153176	1	9,032,844	**3.51E-07**	0.001559	*SYNJ2*	In	Inositol phosphate dephosphorylation
WU_10.2_3_129122235	3	120,870,358	**7.72E-07**	0.060399	*FAM49A*	Up	NA
MARC0081527	6	80,617,330	0.000432722	**3.20E-07**	*C1QB*	Down	Complement activation, classical pathway
WU_10.2_14_389214	14	217,583	**5.23 E-08**	8.22E-06	***SPIN1***	Up	Wnt signaling pathway
WU_10.2_2_12776809	2	13,143,791	0.000571848	**3.33E-07**	***CTNND1***	Up	Negative regulation of canonical Wnt signaling pathway
WU_10.2_6_85867859	6	92,797,244	**6.21E-07**	0.000352248	***GRIK3***	Up	Glutamate receptor signaling pathway
ALGA0012962	2	32,757,436	0.00064956	**4.23E-07**	*LIN7C*	In	Morphogenesis of an epithelial sheet

SNP, single nucleotide polymorphism; SSC, Sus scrofa chromosome; TNB, total number of piglets born; NBA, number of piglets born alive; *LIN7C*, lin-7 homolog C; *LHFPL3*, LHFPL tetraspan submily member 3; *MSX1*, msh homeobox 1; *NECTIN1*, nectin cell adhesion molecule 1; *OR10Q1*, olfactory receptor family 10 subfamily Q member 1; *EYA4*, EYA transcriptional coactivator and phosphatase 4; *GFRA1*, GDNF family receptor alpha 1; *FOXQ1*, forkhead box Q1; *VEGFA*, vascular endothelial growth factor A; *CA8*, carbonic anhydrase 8; *SYNJ2*, synaptojanin 2; *FAM49A*, family with sequence similarity 49 member A; *C1QB*, complement C1q B chain; *SPIN1*, spindlin 1; *CTNND1*, catenin delta 1; *GRIK3*, glutamate ionotropic receptor kainate type subunit 3; VA, not available.

1) p_(meta-TNB)_–value = p-value from the meta analysis. The bold data in this column represent the significant SNP at genome-wide significant level; otherwise at the chromosome-wide significant level.

2) p_(meta-NBA)_–value = p-value from the meta analysis. The bold data in this column represent the significant level.

3) The associated gene in bold in this column represent these genes were associated with traits based on annotation.

4) down/up = the location of SNP in downstream/upstream of the nearest gene.

**Table 4:** The results of multi-trait meta-analysis within four populations

SNP name	SSC	Location (bp)	p_(meta- LM)_–value1)	p_(meta- FJ)_–value2)	p_(meta- XD)_–value3)	p_(meta- ZX)_–value4)	Associated gene5)	Distance6) (bp)	Gene function
ALGA0012964	2	32,799,355	1.04E-06	2.68E-06	1.08E-05	**2.65E-08**	*LIN7C*	In	Morphogenesis of an epithelial sheet
ALGA0054421	9	104,625,702	**1.24E-07**	**6.30E-08**	**4.47E-08**	1.14E-05	***LHFPL3***	In	Self reported educational attainment
ASGA0037579	8	5,703,865	3.01E-06	**3.75E-08**	1.53E-06	**1.37E-07**	***MSX1***	Down	In utero embryonic development
DRGA0000439	1	30,456,989	**3.74E-07**	**3.93E-07**	**3.74E-07**	**6.60E-08**	*EYA4*	In	Protein dephosphorylation
H3GA0021245	7	38,823,945	**1.37E-07**	7.92E-06	**3.82E-07**	**6.41E-09**	***VEGFA***	Down	Regulation of signaling receptor activity
H3GA0042513	14	126,349,106	**2.31E-07**	2.35E-06	**4.84E-07**	1.07E-05	*GFRA1*	In	Nervous system development
MARC0081527	6	80,617,330	**3.34E-08**	**1.45E-07**	**7.55E-09**	7.35E-06	*C1QB*	Down	Complement activation, classical pathway
WU_10.2_14_389214	14	217,583	4.59E-06	**3.02E-08**	1.06E-05	1.64E-06	***SPIN1***	Up	Wnt signaling pathway
WU_10.2_2_12776809	2	13,143,791	**1.57E-08**	2.52E-06	1.99E-06	3.78E-06	*CTNND1*	Up	Negative regulation of canonical Wnt signaling pathway
WU_10.2_6_85867859	6	92,797,244	**5.78E-07**	2.26E-06	4.41E-06	1.53E-06	*GRIK3*	Up	Glutamate receptor signaling pathway
WU_10.2_7_130172562	7	537,474	**1.96E-07**	4.10E-06	1.88E-06	**4.09E-09**	***FOXQ1***	Up	Cell differentiation

SNP, single nucleotide polymorphism; *LIN7C*, lin-7 homolog C; *LHFPL3*, LHFPL tetraspan submily member 3; *MSX1*, msh homeobox 1; *EYA4*, EYA transcriptional coactivator and phosphatase 4; *VEGFA*, vascular endothelial growth factor A; *GFRA1*, GDNF family receptor alpha 1; *C1QB*, complement C1q B chain; *SPIN1*, spindlin 1; *CTNND1*, catenin delta 1; *GRIK3*, glutamate ionotropic receptor kainate type subunit 3; *FOXQ1*, forkhead box Q1.

1)–4) p_(meta-XX)_–value = p-value from the multi-traits meta analysis for four populations LM, FJ, XD, and ZX. The bold data in this column represent the significant SNP at genome-wide significant level; otherwise at the chromosome-wide significant level.

5) The associated gene in bold in this column represent these genes were associated with traits based on annotation.

6) down/up = the location of SNP in downstream/upstream of the nearest gene.
