# Supplementary material for: A genome-wide association study of reproduction traits in four pig populations with different genetic backgrounds
Source: Asian-Australas J Anim Sci. 2019 Oct 21;33(9):1400–10. doi: 10.5713/ajas.19.0411 (PMC7468174; doi:10.5713/ajas.19.0411)
Supplement: Supplementary file 1 [file ajas-19-0411-suppl.pdf]

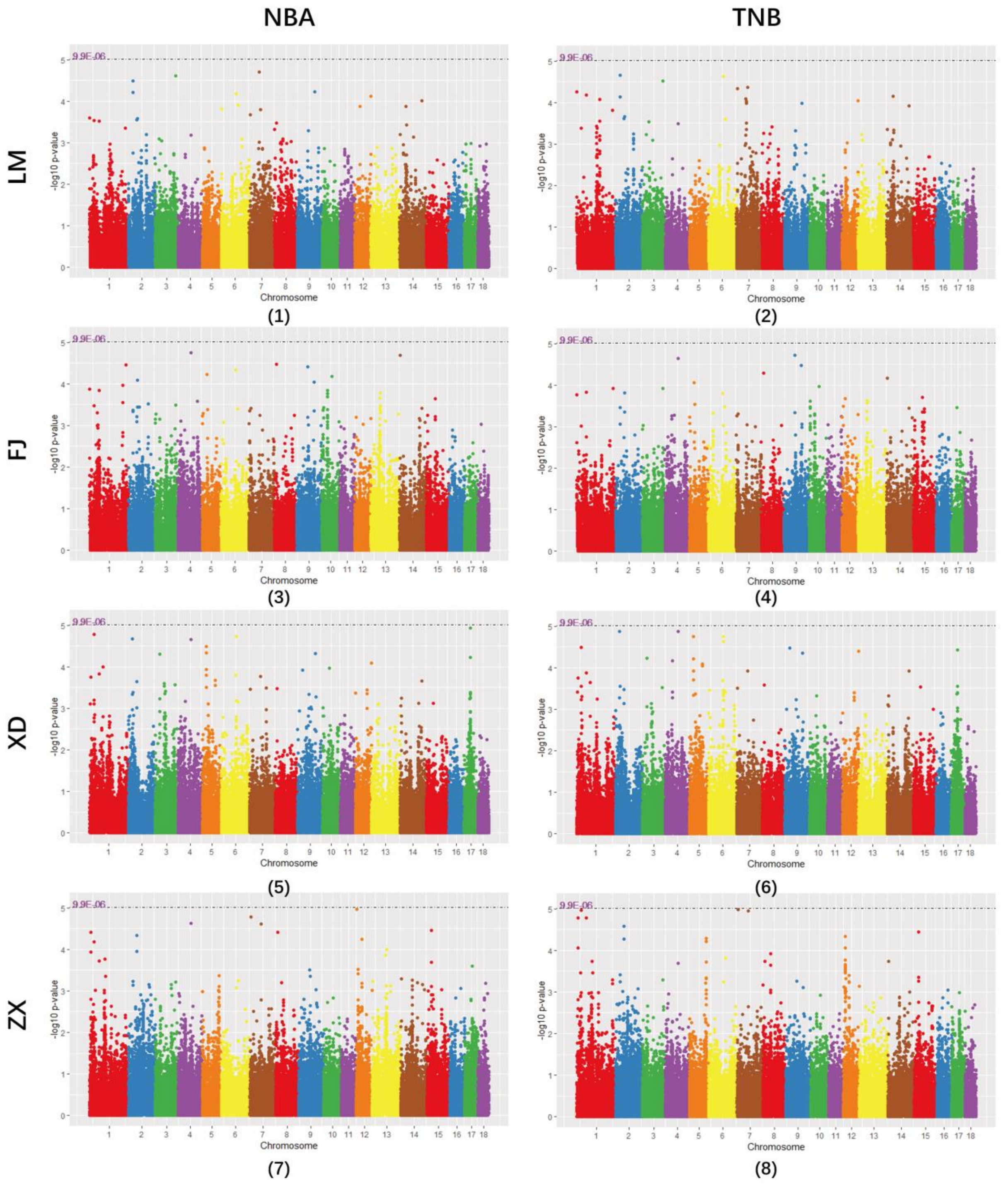

Supplemental Figure S1. Manhattan plot of single trait GWAS for TNB and NBA in four populations. The *x*-axis represents the chromosomes and the *y*-axis represents the $-\log_{10}$(p-value). The dotted line indicates the significance threshold of genome-wide level for GWAS

TNB, total number born; NBA number born alive.

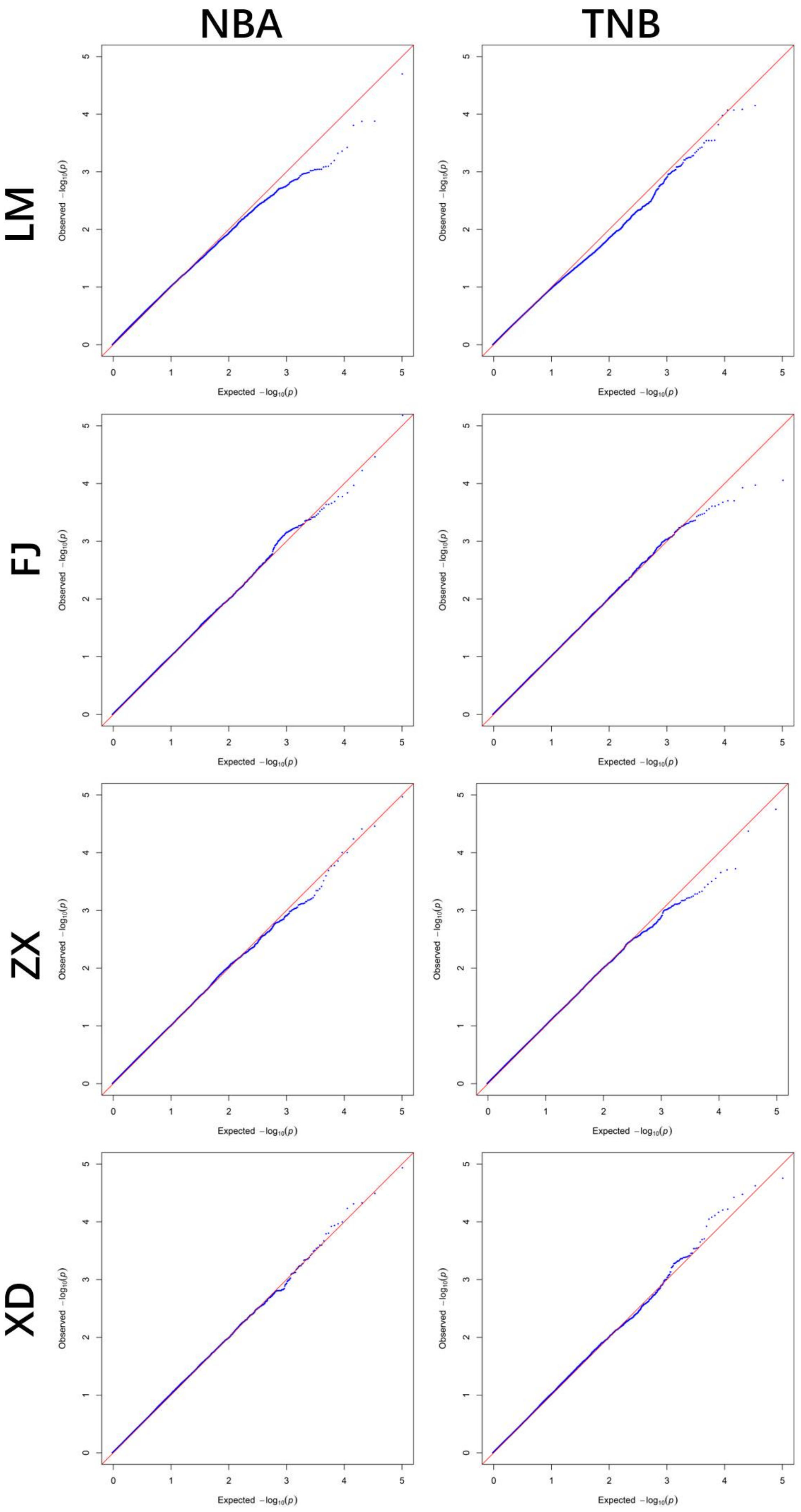
NBA
TNB
LM
FJ
ZX
XD
Observed −log10(p)
Expected −log10(p)
0
1
2
3
4
5

Supplemental Figure S2. Q-Q plots of NBA and TNB in four populations. The *x*-axis and the *y*-axis represent the expected and observed $-\log_{10}$(p-value). TNB, total number born; NBA number born alive.
